# Inspiratory threshold loading negatively impacts attentional performance

**DOI:** 10.3389/fpsyg.2022.959515

**Published:** 2022-09-16

**Authors:** Eli F. Kelley, Troy J. Cross, Bruce D. Johnson

**Affiliations:** ^1^Air Force Research Laboratory (AFRL), 711HPW/RHBFP, WPAFB, Dayton, OH, United States; ^2^Faculty of Medicine and Health, The University of Sydney, Sydney, NSW, Australia; ^3^Department of Cardiovascular Diseases, Mayo Clinic, Rochester, MN, United States

**Keywords:** work of breathing, cognitive performance, reaction time, pilot health, attentional performance

## Abstract

**Rationale:**

There are growing concerns over the occurrence of adverse physiologic events (PEs) occurring in pilots during operation of United States Air Force and Navy high-performance aircraft. We hypothesize that a heightened inspiratory work of breathing experienced by jet pilots by virtue of the on-board life support system may constitute a “distraction stimulus” consequent to an increased sensation of respiratory muscle effort. As such, the purpose of this study was to determine whether increasing inspiratory muscle effort adversely impacts on attentional performance.

**Methods:**

Twelve, healthy participants (age: 29 ± 6 years) were recruited for this study. Participants completed six repetitions of a modified Masked Conjunctive Continuous Performance Task (MCCPT) protocol while breathing against four different inspiratory threshold loads to assess median reaction times (RTs). A computer-controlled threshold loading device was used to set the inspiratory threshold loads. Repeated measures analysis of variances (ANOVAs) were performed to examine: (i) the efficacy of the threshold loading device to impose significantly higher loading at each loading condition; (ii) the effects of loading condition on respiratory muscle effort sensation; and (iii) the influence of hypercapnia on MCCPT scores during inspiratory threshold loading. Generalized additive mixed effects models (GAMMs) were used to examine the potential non-linear effects of respiratory muscular effort sensation, device loading, and hypercapnia, on MCCPT scores during inspiratory threshold loading.

**Results:**

Inspiratory threshold loading significantly augmented (*P* < 0.05) inspiratory effort sensation and the inspiratory pressure-time product (PTP). Our analyses also revealed that median hit RT was positively associated with inspiratory effort sensation during inspiratory loading trials.

**Conclusion:**

The findings of this work suggest that it was not increasing inspiratory muscle effort (i.e., PTP) *per se*, but rather participant’s subjective perception of inspiratory “load” that impacts negatively on attentional performance; i.e., as the degree of inspiratory effort sensation increased, sotoo did median hit RT. As such, it is reasonable to suggest that minimizing inspiratory effort sensation (independent of the mechanical output of the inspiratory muscles) during high-performance flight operations may prove useful in reducing pilot RTs during complex behavioral tasks.

## Introduction

High-performance aircraft are a vital component to a nation’s homeland defense and are a major contributor to air dominance and superiority during joint military (defensive and offensive) operations. These high-performance aircraft are capable of imposing supra-physiologic perturbations on pilots owing to their super cruise capabilities, super-maneuverability, stealth, and embedded/integrated avionics system. Despite the superlative features, it has become apparent over the past decade that these high-performance aircraft are not designed for pilot health, such that flying these aircraft have posed a number of serious concerns for pilot safety, including a constellation of symptoms defined as physiologic events (PEs). The United States Air Force defines a PE as any injury, illness, or abnormal physiological condition experienced by aircrew or others because of the flight environment which may impact pilot health and/or performance. In response to the growing concern over pilot safety, several United States Air Force and Naval investigations and task forces were assembled to identify ways in which to optimize pilot performance and, where possible, eliminate sources of great risk to pilots. In due course, several findings and recommendations were offered, namely those pointing out system-specific factors in the onboard oxygen delivery system. However, in April 2012, the Restrictive Breathing Working Group (RBWG) highlighted a previously unaddressed problem affecting pilot safety: namely that the F-22 Life Support System imposed an excessively high mechanical work of breathing (Committee on Armed Services, 2012). It must not be forgotten, however, that working in the operational aerospace environment, *per se*, imposes unique demands on the respiratory system of the high-performance aircraft pilot that may increase the WOB demands on (Glaister, 1970; Tacker et al., 1987; Estenne et al., 1995; Bain et al., 1997; West, 2013). Taken together, it is evident that breathing is an energetically demanding task for the high-performance aircraft pilot during flight operations. Yet, while it may be clear that a high WOB is implicated in the manifestation of PEs in high-performance aircraft pilots, the precise *mechanisms* by which an elevated WOB impacts on pilot safety remain elusive. We argue below that the perception of increased respiratory muscle effort (consequent to a high WOB) may directly result in performance degradations resulting in Pes by virtue of impaired attentional performance of the high-performance aircraft pilot.

The act of breathing is largely an unconscious experience: rarely do we perceive the muscular effort required to breathe while at rest. However, under circumstances where the mechanical load imposed on the respiratory muscles is elevated, the sensation of breathing effort may increase to the point where it can no longer be ignored (Parshall et al., 2012). Such elevated WOB may be perceived as “increased breathing effort/discomfort,” “air-hunger,” “unsatisfied inspiration,” or “chest tightness” (Parshall et al., 2012; Laviolette and Laveneziana, 2014). Stated in other words, high levels of respiratory muscle work impinge on the consciousness of the individual, and is often experienced as a negative (noxious or pain-related) affective sensation (Morélot-Panzini et al., 2007; Parshall et al., 2012; Laviolette and Laveneziana, 2014). Because elevated respiratory muscle effort may occupy a *non-trivial* portion of the conscious experience, it follows that a requisite amount of cognitive resources must be devoted to “paying attention” to this noxious stimuli (Von Leupoldt et al., 2010). Indeed, previous literature has demonstrated that increased WOB may impact cognitive function (Sharman et al., 2014; Nierat et al., 2016; Sucec et al., 2018; Vinckier et al., 2018). Based on these findings, we suggested that attempts to minimize inspiratory effort sensation (independent of device resistance) during flight operations may prove useful in reducing pilot reaction times (RT) during complex behavioral tasks, especially when only tight margins of error can be tolerated. As such, minimizing inspiratory effort sensation may minimize performance degradations resulting in PEs.

Therefore, the primary objective of this work was to evaluate the impact of increasing inspiratory WOB on attentional performance in healthy adults. We sought to extend upon the findings of previous literature (Sharman et al., 2014; Nierat et al., 2016; Sucec et al., 2018; Vinckier et al., 2018) by recruiting both healthy male and female subjects and stressing their cognitive resources with a Masked Conjunctive Continuous Performance Task (MCCPT), a task used to strain central processing and assess sustained and selective attentional performance (Shalev et al., 2018). The MCCPT was chosen as it requires one to rapidly interpret shapes and colors and make appropriate decisions based on these interpretations, similar to the complex decision making that high-performance aircraft undertake. As such, the novelty of the current study when compared to previous research was our ability to demonstrate the influence of increased WOB on attentional performance in healthy male and female subjects while completing a complex decision-making task. Furthermore, the inclusion of P_*ET*_O_2_ and P_*ET*_CO_2_ provides insight into the effect threshold breathing may have on end-tidal gases and how changes in P_*ET*_O_2_ and P_*ET*_CO_2_ may impact attentional performance. We hypothesized that under circumstances of increased inspiratory threshold loading, the augmented perception of inspiratory muscle effort would compete for available cognitive resources, impairing subjects’ attentional performance.

## Materials and methods

### Participants

Twelve healthy male participants (age: 29 ± 6 years) were recruited for this study. Participants had no known history of cardiac, pulmonary, and/or metabolic disease, and no reported mental or psychological disorders of attention. Each participant completed two visits to the laboratory. The present study conformed to the principles outlined in the Declaration of Helsinki and was approved by the Mayo Clinic Internal Review Board.

### Measured and computed variables

#### Masked conjunctive continuous performance scores

To assess continuous attentional performance, we used the MCCPT – the psychometric tool we used to strain central processing and assess sustained and selective attentional performance (Shalev et al., 2018). In short, a colored mask comprised of four superimposed shapes of different color (circle, square, triangle, and hexagon) was presented on screen. To avoid habituation effects, two mask images of differing outline thickness were alternated every 10−20 ms. The mask was removed to reveal either the target (i.e., red circle) or a distractor (i.e., any other combination of shape and color). Additionally, the inter-stimulus interval was randomly jittered between 2,000 and 5,000 ms. The MCCPT was chosen because we felt that by occupying/requiring a greater fraction of participant’s attentional focus, we may engender larger impacts on RTs as we progressively increased inspiratory threshold loads. Additionally, we reasoned the MCCPT more closely aligns with the complex decision making that high-performance aircraft pilots encounter – they are required to expeditiously interpret shapes and colors and make appropriate decisions based on these interpretations.

The principal measurement used in the computation of the continuous performance scores of the MCCPT is participants’ RT in response to the stimulus presentation (i.e., target or distractor). The accurate measurement of RT is therefore dependent on the degree of accuracy with which both stimulus presentation and the responding mechanical keypress can be recorded. To this end, our custom-built microcontroller device measured the onset of stimulus presentation *via* a light sensor attached 14 to the LCD computer display. The mechanical keypress was readily detected as a switching state from high to low on a digital input port of the microcontroller. The time elapsed between these two events was measured *via* an interrupt-driven routine that was able to provide elapsed durations with sub millisecond accuracy. The microcontroller device communicated with a host PC *via* USB, such that trial correctness could be matched with the RT measured by the microcontroller device. Each stimulus presentation was coded into one of the following two categories, depending on the shape and color of the stimuli:

Conjunctive = Stimuli with the same shape or color to the target

• Color Conjunctive = Stimuli with the same color as the target
• Shape Conjunctive = Stimuli with the same shape as the target

Non-conjunctive = Stimuli with a different shape and/or color to the target.

Only correct responses were used in the calculation of hit RT values. RT values were excluded from analysis if the observed value was <200 or ≥1,000 ms. This was done as RTs < 200 ms were considered either a “false start” or a late response to the previous stimuli and RTs ≥ 1,000 ms were defined as a non-response or lapse in attention. It is important to note, that no responses were outside of these RT limits (e.g., the fastest observed RT was 285 ms). The resulting distributions of RT values for each participant were typically non-normal and, as such, median RTs for the above conditions were computed for each loading condition, separately. Additional parameters computed were error types (i.e., commission and omission); the ability to discriminate the target from the distractor (d′), which incorporates the two error types – commissions and omissions; and the criteria (β) which provides a measure of the balance between error types.


$${\text{d}}^{\prime }=\text{z}\,\left(\mathrm{hit}\,\mathrm{rate}\right)-\text{z}\,\left(\mathrm{false}\,\mathrm{alarm}\,\mathrm{rate}\right)$$

β=Covariance/Variance

Omission errors refer to a subject not responding to a stimulus the subjects *was* supposed to (e.g., not responding to a red triangle) whereas a commission error refers to a subject responding to a stimulus the subject was *not* supposed to (i.e., responding to a red circle). For β, a positive value means a higher tendency toward omission errors, and vice versa (when β value is zero, there is no bias toward any error type).

#### Inspiratory pressures

Mouth pressure was sampled *via* a lateral port located in the mouthpiece and connected to a differential pressure transducer (HSCSNDN005PDAA5, Honeywell International Inc., NJ, United States). Inspiratory and expiratory flows were measured separately using heated pneumotachographs (3813 series, Hans Rudolph, KS, United States). A humidifier was arranged in-series with the inspiratory limb of the circuit such that the inspired air had an approximate humidity of 100%. Inspiratory muscle effort was expressed as the pressure-time product (PTP) which was quantified as the product of the average inspiratory mouth pressure (P_*i*,*avg*_) and the duration of inspiration (T_*i*_).

#### End-tidal gases, pulse oximetry, and heart rate

The partial pressures of O_2_ and CO_2_ (P_*ET*_O_2_ and P_*ET*_CO_2_, respectively) were measured *via* a rapid-response O_2_/CO_2_ analyzer (GA-200B, iWorx, NH, United States) from a sample line placed in the expiratory limb of the experimental breathing circuit. Pulse oxygenation was measured *via* the fingertip of the non-dominant hand (Radical 7, Massimo, CA, United States). Heart rate and rhythm was recorded using a single-channel bio-amplifier module (FE132, ADInstruments, NSW, Australia).

### Experimental design

To determine the impact of increasing the inspiratory threshold effort on attentional performance, participants visited the laboratory on two separate occasions. During the first visit (Visit 1), pulmonary function testing was performed, and participants were familiarized with the MCCPT. For this testing, we developed a novel microcontroller-based device to implement the MCCPT – the device provided RT values with a sub-millisecond accuracy. The original version of the MCCPT, as developed by Shalev et al. (2018), takes approximately 20 min to complete. However, not all participants may be capable of tolerating a given inspiratory threshold load for this length of time. This point is particularly important given that it was our intention to examine attentional performance across various, and potentially heavy loads. Thus, we modified the original long version of the MCCPT by dividing the protocol into six 21/2 min sequences (∼40 stimulus presentations in each trial). In this manner, we could apply a given threshold load for a relatively brief duration of time, and, through the six repetitions, we were able to accumulate the necessary number of stimulus responses to compute the RT and error rate scores as per the original long version of the MCCPT.

During Visit 2, participants breathed on a two-way non-rebreathing valve to separate the inspiratory and expiratory circuits. Inspiratory and expiratory flows were measured separately using heated pneumotachographs (3813 series, Hans Rudolph, KS, United States). A humidifier was arranged in-series with the inspiratory limb of the circuit. A computer-controlled adjustable poppet valve was interposed between the inspiratory port of the two-way non-rebreathing valve and the humidifier. This valve was adjustable *via* a custom-built software interface and was used to set the threshold load during each trial. The participant was instructed to complete 24 trials of the MCCPT protocol (40 visual stimuli per trial). During each trial of this MCCPT protocol, one of four loads were added to the inspiratory circuit in such a way that the peak inspiratory mouth pressure achieved either <5, ∼10, ∼20, or ∼40% of the recorded baseline maximal inspiratory pressures (MIPs), notated here as loads 1 (control), 2, 3, and 4. These loads were imposed in a blinded manner in randomized order such that six repetitions of each load were presented across the course of the visit (i.e., 24 total trials). These load levels were chosen because pilot testing revealed that most subjects would be capable of maintaining ∼40% of the recorded baseline MIP without coming off the mouthpiece for the duration of the 21/2 min trial. Given this upper limit for “tolerable” inspiratory threshold loading in our cohort, we set the remaining three loads at <5, ∼10, and ∼20% of baseline MIP. In doing so, we were able to ensure that, even with minimal variability in peak mouth pressure, the data would still yield four distinct load levels.

Immediately after each trial was completed, the participant was asked to rate their perceived inspiratory muscle effort required to breathe against the imposed load on the modified 10-point category ratio scale (CR10; Mahler and Horowitz, 1994). Approximately 2 min of rest was given between each trial. Participants wore noise-canceling headphones to reduce the effects of ambient noise and environmental distraction on their MCCPT performance. Every six trials, the participant was given a longer break (∼10 min) where they were taken off the mouthpiece and were free to move and walk around. During each six-trial run, however, participants were instructed to remain on the mouthpiece. MIPs were obtained at the end of each six-trial run to assess whether inspiratory muscle fatigue was evident. MIP testing was conducted according to American Thoracic Society (ATS) and the European Respiratory Society (ERS) recommendations (Society and Society, 2002; Laveneziana et al., 2019).

Due to the nature of the MCCPT, we could not provide any visual or auditory feedback on breathing pattern and respiratory muscle effort for fear of distracting the participant from the task at hand. As such, participants were free to adopt any rate or depth of breathing they felt most comfortable during loading conditions 1–4. The four loads were determined before experimental data collection began, on a participant-by-participant basis. The investigator varied the load imposed by the computer-controlled variable threshold device until the peak mouth pressure swing was approximately 10% of the recorded baseline MIP – this condition was set as load 2. Loads 3 and 4 were determined in similar fashion by adjusting the threshold loading valve until peak inspiratory mouth pressure swings were ∼20 and ∼40% of baseline MIP, respectively. Load 1 was set at minimal load with the poppet valve fully disengaged and thus making no contact with the valve seat (e.g., no threshold load). It is emphasized that the determination of each load (1 through 4) was performed while the participant was practicing the MCCPT protocol. Because participants were spontaneously breathing during the loaded trials (i.e., no feedback was given), the peak mouth pressure swings for a given “intended” load were liable to change slightly over the course of the 24 experimental trials. Hence, it was sometimes necessary to adjust the threshold load at a given condition to bring the peak inspiratory mouth pressure swing back into the desired range. Although a rare occurrence, if any adjustments were necessary, they were performed *between* and not *during* trials.

### Statistical analyses

The measured and computed variables obtained during the six repetitions of each load were averaged to provide a single value per load, per subject. Repeated measures analyses of variance (ANOVAs) were used to determine the effect of increasing threshold load (Glaister, 1970; Tacker et al., 1987; Estenne et al., 1995; Committee on Armed Services, 2012) on respiratory mechanics, breathing pattern, end-tidal gases, and MCCPT performance.

Generalized additive mixed effects models (GAMMs) were used to determine the impact of respiratory mechanics, respiratory effort sensation, and end-tidal partial pressure of CO_2_ (P_*ET*_CO_2_) on MCCPT performance scores. The parameter of respiratory mechanics that was chosen as a covariate in these GAMM models was the empirically determined inspiratory PTP generated in response to the load imposed by the adjustable poppet valve during each loading condition. We opted for this parameter because it can be more readily obtained under operational conditions (i.e., cockpit) compared with those variables that require instrumentation with esophageal and gastric balloon catheters and measurement of esophageal pressure (P_*es*_) (e.g., WOB, etc.). Additionally, P_*ET*_CO_2_ and P_*ET*_O_2_ were calculated as a change from baseline to account for the variability in subject resting end-tidal partial pressures. Statistical significance was considered if *P* < 0.05.

The GAMM models used in this study were selected through the interrogation of multiple competing models. Additionally, competing distributional families were compared using the Akaike information criterion (AIC) to determine which family was most appropriate. Through these comparisons, we determined a log linked Gamma distribution most closely fit the data for the reaction time models, and a beta regression family most closely fit the data for the error rate models. Included in these models were main effects and random effects for patient ID, respiratory effort sensation, PTP, P_*ET*_CO_2_, and P_*ET*_O_2_. It is important to note that while hyper- and hypocapnia have been associated with altered cognitive performance, there are conflicting reports as to their relationship (i.e., positive or negative) (Sayers et al., 1987; Bloch-Salisbury et al., 2000; Thesen et al., 2012). The presence of altered P_*ET*_CO_2_ and the current uncertainty of how altered arterial CO_2_ may affect cognition, were driving forces behind our decision to include P_*ET*_CO_2_ in our GAMM models.

The selection of group-level main effects and interaction terms was determined using a backward selection method based on the AIC score (Wood, 2017). The final GAMM model was fit using the restricted maximal likelihood (REML) method, cubic regression penalties for non-linear smooths, the hyperparameter γ was calculated using BIC-like parameters [i.e., log(*n*)/2] to reduce overfitting, and a false discover *P*-value adjustment to reduce false positives (Wood, 2008; Reiss and Todd Ogden, 2009). An extra penalty was added to each individual term so it could be penalized to zero, thereby allowing terms to be automatically “selected out” from the GAMM when appropriate.

## Results

### Inspiratory mouth pressures, pressure-time product, effort sensation, and ventilatory parameters

**FIGURE 1:**
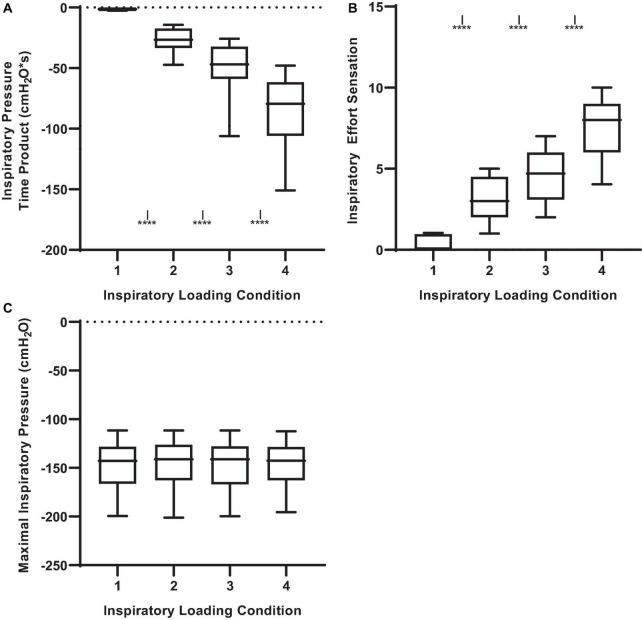
Inspiratory pressure-time product, effort sensation and maximal inspiratory pressure (MIP) across inspiratory threshold loading conditions during masked conjunctive continuous performance trials. **(A)** Depicts the differences in inspiratory pressure time product across loading conditions. **(B)** Depicts the differences in Inspiratory effort sensation across loading conditions. **(C)** Depicts the differences in maximal inspiratory pressure across loading conditions. The upper and lower limits of the box represent the 95% CI, the horizontal line in the box represents the mean, and the whiskers represent the SEM. ****Significant difference from previous load condition, *P* < 0.001. *^I^*Significant difference from load condition 1, *P* < 0.05.

**FIGURE 2:**
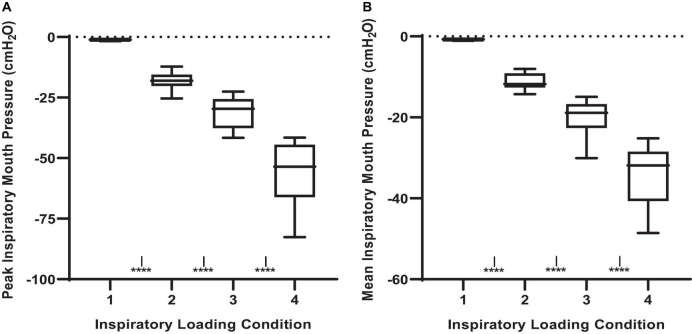
Peak and mean inspiratory mouth pressure swings across inspiratory threshold loading conditions during masked conjunctive continuous performance trials. **(A)** Depicts the differences in peak inspiratory mouth pressure across loading conditions. **(B)** Depicts the differences in mean inspiratory mouth pressure across loading conditions. The upper and lower limits of the box represent the 95% CI, the horizontal line in the box represents the mean, and the whiskers represent the SEM. ****Significant difference from previous load condition, *P* < 0.0001, *^I^*Significant difference from load condition 1, *P* < 0.05.

The inspiratory PTP, and inspiratory effort sensation, together increased with augmenting inspiratory loads (Figures 1A,B; *P* < 0.001). The peak and mean inspiratory mouth pressure swings during the inspiratory threshold loading conditions are presented in Figure 2. There was a load-dependent rise in the magnitude of peak inspiratory mouth pressure swings (*P* < 0.001) (Figure 2A). Furthermore, there was a similar pattern of increasing mean inspiratory mouth pressure was observed with augmenting inspiratory threshold loads (*P* < 0.001). These observations are important because they serve to confirm that, on average, we successfully maintained the participant’s spontaneous peak inspiratory mouth pressure swings within the “intended” ranges for each loading condition. Taken together, these findings confirm that inspiratory muscle pressure-development was progressively increased in response to the rising threshold loads. Although the imposed threshold loads were indeed large, our participants did not appear at risk of developing inspiratory muscle fatigue – an observation which corroborates the stable MIP values observed across loading conditions (Figure 1C). We are thus confident that our approach to determining, and imposing the four different loads did, in fact, engender separate inspiratory threshold pressures and evoked unique increases in the sensation of inspiratory muscle effort.

**FIGURE 3:**
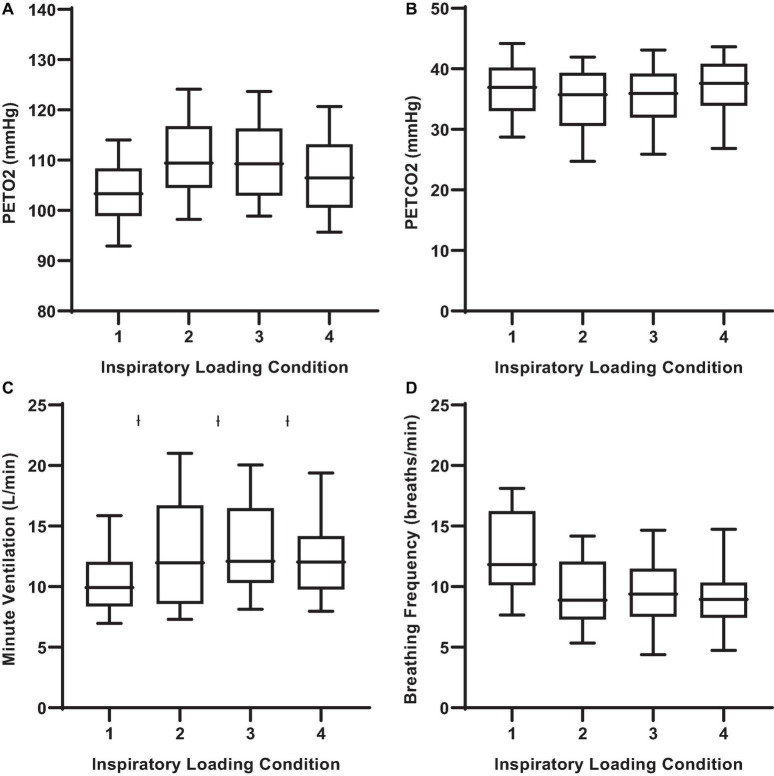
End-tidal gases and ventilatory responses to inspiratory threshold loading during masked conjunctive continuous performance trials. **(A)** Depicts the differences in PETO2 across loading conditions. **(B)** Depicts the differences in PETCO2 across loading conditions. **(C)** Depicts The differences in minute ventilation across loading conditions. **(D)** Depicts changes in breathing frequency across loading conditions. The upper and lower limits of the box represent the 95% CI, the horizontal line in the box represents the mean, and the whiskers represent the SEM. P_*ET*_O_2_ and P_*ET*_CO_2_: end-tidal partial pressures of O_2_ and CO_2_. *^I^*Significant difference from load condition 1, *P* < 0.05.

Given that participants were free to adopt any rate and depth of breathing during the MCCPT trials, there was a certain degree of variability in the breathing pattern response to inspiratory threshold loading. Nonetheless, there were some observable trends that were mostly evident at the higher levels of inspiratory resistance (e.g., loads 2 and 3). Minute ventilation increased with augmenting inspiratory load (Figure 3). This increased minute ventilation was the result of increases in tidal volume rather than breathing frequency wherein tidal volume was increased in conditions 2, 3, and 4 when compared to load 1 (*P* ≤ 0.001). Interestingly, this relative hyperpnea was not accompanied by significant changes in P_*ET*_CO_2_ nor P_*ET*_O_2_ (Figure 3).

### Masked conjunctive continuous performance task results during inspiratory loading

**FIGURE 4:**
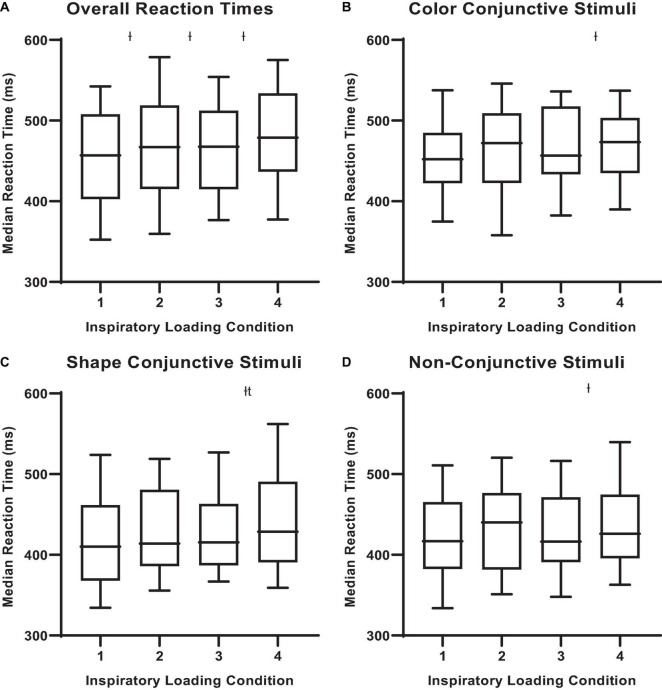
Median reactions times for different stimuli across inspiratory threshold loading conditions during masked conjunctive continuous performance trials. **(A)** Depicts the differences in overall median reaction times across loading conditions. **(B)** Depicts the differences in median reaction times for color conjunctive stimuli across loading conditions. **(C)** Depicts the differences in Median Reaction Times for shape conjunctive stimuli across loading conditions. **(D)** Depicts the differences in median reaction times for non-conjunctive stimuli across loading conditions. The upper and lower limits of the box represent the 95% CI, the horizontal line in the box represents the median, and the whiskers represent the SEM. *^I^*Significant difference from load condition 1, *P* < 0.05. *^t^*Significant difference from load condition 2, *P* < 0.05.

**FIGURE 5:**
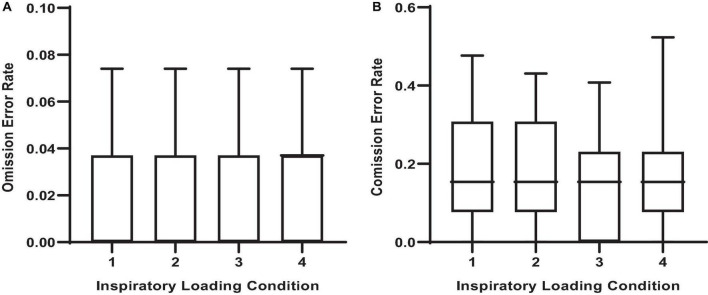
Error rates across inspiratory threshold loading conditions during masked conjunctive continuous performance trials. **(A)** Depicts the differences in omission error rates across loading conditions. **(B)** Depicts the differences in commission error rates across loading conditions. The upper and lower limits of the box represent the 95% CI, the horizontal line in the box represents the mean, and the whiskers represent the SEM. There were no differences in error rates between loading conditions.

There were observable changes in MCCPT performance across inspiratory threshold loading conditions – wherein median hit RT was significantly longer during loads 2, 3, and 4 compared with load 1 (Table 1 and Figure 4; *P* < 0.05). However, there were no differences in omission nor commission error rates between the inspiratory loading conditions (Figure 5). While there were no differences in the overall error rates, how the stimuli were presented did influence RT – wherein median hit RT during load 4 was significantly higher for color conjunctive (*P* = 0.031), shape conjunctive (*P* < 0.001), and non-conjunctive (*P* < 0.01) distractor stimuli when compared to load 1. Furthermore, median hit RT was higher for the shape conjunctive distractor stimuli when compared to load 2 (*P* = 0.012).

**TABLE 1:** Median hit reaction times across inspiratory threshold loading conditions.

Loading conditions			

Condition	Median (ms)	SD	*P*-value
1 (no load)	441.4	81.9	
2 (light load)	468.3	80.6	**0.049**
3 (moderate load)	471.4	83.9	**0.024**
4 (heavy load)	481.7	108.6	**0.002**

SD, standard deviation. Bolded *P*-values denote a significant difference between the inspiratory and load and load 1 (*P* < 0.05).

### Non-linear trends in reaction times during inspiratory threshold loaded breathing

**FIGURE 6:**
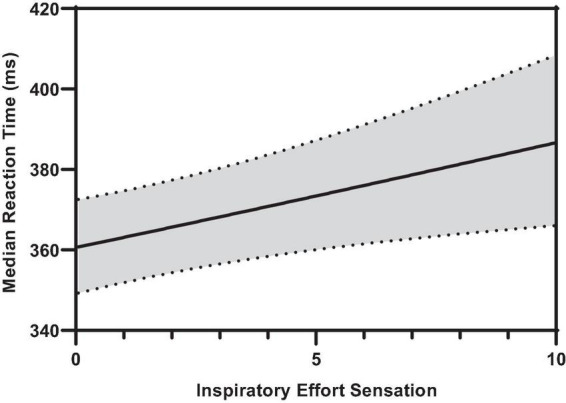
Predicted smooth in median reaction time across inspiratory effort sensation. The predicted smooth of median reaction time was obtained from generalized additive mixed effects modeling (GAMM) of measures of median reaction time where inspiratory effort sensation, inspiratory PTP, and percent change in P_*ET*_O_2_, and P_*ET*_CO_2_ were entered into the model as covariates. The predicted smooth curve was produced for median reaction time by setting all other covariates at their means (inspiratory PTP = –32.65 cmH_2_O⋅s; ΔP_*ET*_O_2_ = 7.31%; ΔP_*ET*_CO_2_ = –4.04%). The dashed curves show the 95% CIs around the prediction.

Figure 6 illustrates the effect of inspiratory effort sensation on overall median hit RT. Increased inspiratory effort sensation had deleterious effects on the overall, color conjunctive, and shape conjunctive median hit reaction times (Table 2 and Supplementary Table 1). Across all stimuli types (e.g., non-conjunctive, color conjunctive, etc.), as inspiratory effort sensation increased, RT increased as well. Interestingly, although there was no main effect of inspiratory PTP on overall median hit RT, inspiratory PTP was negatively associated with both color conjunctive stimuli and non-conjunctive stimuli. Furthermore, there were no main effects of P_*ET*_O_2_ or P_*ET*_CO_2_ for overall, color conjunctive, shape conjunctive, and non-conjunctive median hit RT.

**TABLE 2:** Generalized additive mixed effects model (GAMM) results for overall median hit reaction time during inspiratory loaded trials.

	Estimate*	SE	Statistic	*P*-value
**Main effects**				
Intercept	6.07	0.02	283.30	**<0.001**
s(effort sensation)	0.79	–	6.65	**<0.001**
s(pressure-time product)	<0.01	–	0.00	0.13
s(ΔPETCO2)	<0.01	–	0.00	0.57
s(ΔPETO2)	<0.01	–	0.00	0.16
**Random effects**				
ID	0.11	–	99.63	**<0.001**
ID:effort sensation	<0.01	–	0.00	**<0.05**
ID:pressure-time product	<0.01	–	0.00	0.25
ID:ΔPETCO2	<0.01	–	0.00	0.29
ID:ΔPETO2	<0.01	–	0.00	0.29

SE, standard error. Statistic refers to the *T*-value for the intercept and the *F*-value for the smooth terms and random effects; pressure-time product was measured in cmH_2_O × min; ΔP_*ET*_CO_2_ and ΔP_*ET*_O_2_ were measured in mmHg; bolded *P*-values denote a significant influence of the covariate term on overall median hit reaction times (*P* < 0.05). *For all smooth terms [s()] this estimate represents the estimated degrees of freedom of the corresponding smooth.

### Error rates during inspiratory threshold loaded breathing

**FIGURE 7:**
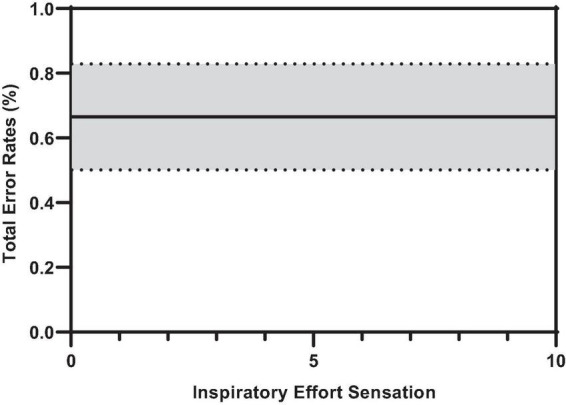
Predicted smooth in total error rates across inspiratory effort sensation. The predicted smooth of total error rates was obtained from generalized additive mixed effects modeling (GAMM) of measures of total error rates where inspiratory effort sensation, inspiratory PTP, and percent change in P_*ET*_O_2_, and P_*ET*_CO_2_ were entered into the model as covariates. The predicted smooth curve was produced for total error rates by setting all other covariates at their means (inspiratory PTP = –32.65 cmH_2_O⋅s; ΔP_*ET*_O_2_ = 7.31%; ΔP_*ET*_CO_2_ = –4.04%). The dashed curves show the 95% CIs around the prediction.

**FIGURE 8:**
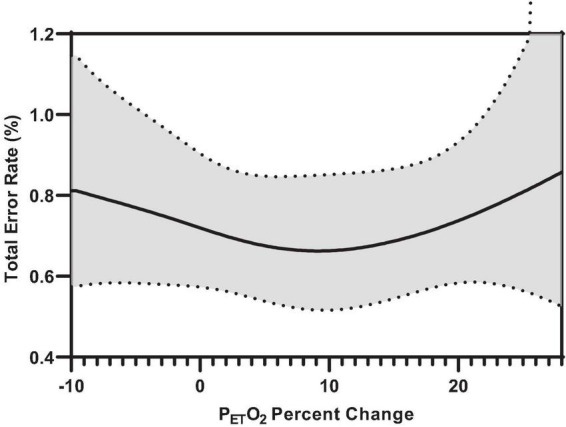
Predicted smooth in total error rates for percent change P_*ET*_O_2_ from baseline. The predicted smooth of total error rates was obtained from generalized additive mixed effects modeling (GAMM) of measures of total error rates where inspiratory effort sensation, inspiratory PTP, and percent change in P_*ET*_O_2_, and P_*ET*_CO_2_ were entered into the model as covariates. The predicted smooth curve was produced for total error rates by setting all other covariates at their means (inspiratory effort sensation = 3.89; inspiratory PTP = –32.65 cmH_2_O⋅s; ΔP_*ET*_CO_2_ = –4.04%). The dashed curves show the 95% CIs around the prediction.

Figure 7 illustrates the effect of inspiratory threshold loading on total error rates. There was no main effect of inspiratory effort sensation on total error rates (Table 3). Further, there were no main effects of PTP or the change in P_*ET*_CO_2_ from baseline. On the other hand, there was a main effect of the percent change in P_*ET*_O_2_ on total error rates (Figure 8). These data suggest a small increase in P_*ET*_O_2_ (up to approximately a 10% increase from baseline) may decrease (i.e., improve) total error rates but an increase in P_*ET*_O_2_ beyond that ∼10% threshold increases error rates. Conversely, a decrease in P_*ET*_O_2_ from baseline is associated with an increase in total error rates.

**TABLE 3:** Generalized additive mixed effects model (GAMM) results for total error rates during inspiratory loaded trials.

	Estimate*	SE	Statistic	*P*-value
**Main effects**				
Intercept	−0.93	0.11	−8.52	**<0.001**
s(effort sensation)	<0.01	–	0.00	0.14
s(pressure-time product)	<0.01	–	0.00	0.94
s(ΔP_*ET*_CO_2_)	<0.01	–	0.00	0.65
s(ΔP_*ET*_O_2_)	1.00	–	18.29	**<0.01**
**Random effects**				
ID	0.10	–	526.47	**<0.001**
ID:effort sensation	<0.01	–	0.00	0.13
ID:pressure-time product	<0.01	–	0.00	0.79
ID:ΔP_*ET*_CO_2_	<0.01	–	0.00	0.33
ID:ΔP_*ET*_O_2_	<0.01	–	0.00	0.07

SE, standard error. Statistic refers to the *T*-value for the intercept and the *F*-value for the smooth terms and random effects; pressure-time product was measured in cmH_2_O × min; ΔP_*ET*_CO_2_ and ΔP_*ET*_O_2_ were measured in mmHg; bolded *P*-values denote a significant influence of the covariate term on overall median hit reaction times (*P* < 0.05). *For all smooth terms [s()] this estimate represents the estimated degrees of freedom of the corresponding smooth.

When total error rates were stratified by error type, we observed a similar relationship between percent change in P_*ET*_O_2_ and commission errors (responding to a stimulus the participant was *not* supposed to) (Supplementary Table 2). Specifically, an increase or decrease in P_*ET*_O_2_ from the approximate baseline may decrease or improve commission error rates during inspiratory threshold loaded breathing. Further, there were no main effects of effort sensation, PTP, or P_*ET*_CO_2_ on commission error rates. Additionally, there was a main effect of PTP on omission error rates (not responding to a stimulus the subject *was* supposed to) (Supplementary Table 2).

## Discussion

While the act of breathing is largely an unconscious experience, under circumstances of increased mechanical load imposed on the respiratory muscles, the sensation of breathing effort may increase to the point where it can no longer be ignored (Parshall et al., 2012; Laviolette and Laveneziana, 2014). In such a case, high levels of WOB may impose an influence on the consciousness of the individual as a noxious affective sensation (Morélot-Panzini et al., 2007; Parshall et al., 2012; Laviolette and Laveneziana, 2014). Therefore, it is reasonable that this noxious stimuli may occupy a portion of cognitive resources (Von Leupoldt et al., 2010). While previous literature has shown a correlation between dyspnea and cognitive impairments (Dodd et al., 2010; Irani et al., 2017), experimental evidence of the underlying mechanisms are lacking (Sucec et al., 2018). To this end, recent research has demonstrated that increased WOB may have a direct impact on cognitive function (Sharman et al., 2014; Nierat et al., 2016; Sucec et al., 2018; Vinckier et al., 2018). Specifically, inspiratory loading may activate motor respiratory-related cortical networks, leading to diminished cognitive performance (Taytard et al., 2021). Indeed, pilot work by our group suggests increasing inspiratory effort sensation may prolong the period of central information processing during complex reaction time tasks. We argued that an increased perception of inspiratory muscle effort may directly impair attentional performance of the jet fighter pilot. The findings of this preliminary report (Cross and Bruce, 2018) were that central processing speed (reaction time) progressively lengthened (worsened) the greater that inspiratory muscle “effort” sensation increased across the loading trials. Moreover, there was a tendency for error-rate to increase (decrease in response accuracy) at the highest of the resistive loads (∼100 cmH_2_O/L/s). In consideration to previous literature, we hypothesized that increased inspiratory muscle effort sensation, owing to increased WOB, would compete for available cognitive resources, impairing subjects’ attentional performance.

The present work examined the effects of inspiratory threshold loading on attentional performance, as measured *via* a modified version of the MCCPT. Our findings are consistent with previous literature (Sharman et al., 2014; Nierat et al., 2016; Sucec et al., 2018; Vinckier et al., 2018) insofar as they demonstrate that augmenting inspiratory effort sensation may delay central processing during complex behavioral tasks. Furthermore, these data highlight that the *perception* of the inspiratory load is the primary factor influencing overall median hit RT, not the quantitative amount of respiratory muscular power (i.e., effort) expended during inspiration. Specifically, as a participant’s perception of their inspiratory muscle effort increased, their central processing (i.e., RT) was delayed. Thus, we believe the perception of inspiratory effort may indeed compete for a portion of attentional resources that would otherwise be devoted to the continuous engagement and vigilance required by the MCCPT, thereby stressing attentional performance to the point of delayed processing time. To this end, augmenting inspiratory effort sensation, independent of actual inspiratory load, may prolong RT. Specifically, one could expect that median RT would increase by approximately 26 ms as inspiratory effort sensation rises from 0 to 10 respectively. It is important to note that these increases in RT constitute delays in processing and reaction for a single task. From an operational perspective, a 120-min flight where the pilot performs 10 tasks/min constitutes 1,200 total tasks during flight. Were a pilot’s central processing delayed by an average of 20 ms, the total reaction time delay during this 120-min flight would be roughly 24 s – an order of magnitude that may prove operationally relevant for the high-performance aircraft pilot. The current study also demonstrates that when high inspiratory threshold loads were imposed, participants had difficulty identifying the target and discriminating it from the distractors. The impaired ability to discriminate between target and distractors is demonstrated by the increased median RT for color, shape, and non-conjunctive stimuli as inspiratory threshold loads were increased. These findings support our original hypothesis, in that breathing on high inspiratory loads may stress attentional resources preventing the participants from continuously engaging in the MCCPT, thus diminishing perception and delaying RT (Müller and Humphreys, 1991).

Our findings also demonstrated that larger inspiratory threshold loads may alter ventilatory patterns but not to such an extent as to significantly influence end-tidal gases, a finding consistent with the literature (Yanos et al., 1990; Eastwood et al., 1994). However, further interrogation of the non-linear models revealed an influence of P_*ET*_O_2_ on attentional performance. Specifically, the data demonstrated an influence of P_*ET*_O_2_ on error rates during inspiratory threshold loading trials. Specifically, we demonstrated a small increase in P_*ET*_O_2_ from baseline may induce a “hypervigilant state” wherein participants were (slightly) more capable of maintaining continuous engagement in the task. This is supported by the improvements in target and distractor discrimination. However, a >10% increase or decrease in P_*ET*_O_2_ from baseline was associated with increased error rates. Interestingly, increased alveolar O_2_ tension is generally thought to cause cerebral vasoconstriction, resulting in decreased cognitive performance (Smith et al., 2016; Shykoff and DiPasquale, 2018). However, recent literature suggests that poikilocapnic hyperoxia may increase cerebral blood flow, thus improving cognitive function (Smith et al., 2016). As such, an increased P_*ET*_O_2_ in combination with fluctuating P_*ET*_CO_2_ may indeed improve cerebral blood flow and cognitive function. These data suggest alterations in P_*ET*_O_2_ may modulate cerebral blood flow and affect one’s ability to discriminate between the “target” and “non-target” stimuli, resulting in increased error rates (Young et al., 1991; Poulin et al., 1996). It must be mentioned, that given the relatively well maintained P_*ET*_O_2_ levels observed across all conditions, these results may be confounded by a general sparsity of data above and below baseline P_*ET*_O_2_ levels (Figure 3), or that only within a small range of P_*ET*_O_2_ values was the probability of an error lower than “chance.” These data suggest maintaining P_*ET*_O_2_ within a narrow range may improve attentional performance. However, given that we are observing an effect of slightly increasing an already *really small* error rate (<1%), the operational relevance remains unclear.

Our findings extend upon those provided by Vinckier et al. (2018) among others (Sharman et al., 2014; Nierat et al., 2016; Sucec et al., 2018) in that we recruited healthy subjects of both genders and stressed their cognitive resources during graded inspiratory threshold loads while performing a complex decision-making cognitive task. In doing so, we demonstrated that increased inspiratory effort sensation (owing to increased WOB) may impact attentional performance in healthy male and female subjects. Based on these findings, we suggest that attempts to minimize inspiratory effort sensation (independent of device resistance) during flight operations may prove useful in reducing pilot reaction times during complex behavioral tasks, especially when only tight margins of error can be tolerated.

## Conclusion

These data support our hypothesis that imposing a “distraction” *via* increased inspiratory effort sensation leads to impairments in cognitive performance within the domain of attentional focus. Taken together, the current data demonstrates minimizing inspiratory effort sensation and maintaining end-tidal gas concentrations relative to baseline may serve to preserve attentional performance. In an operational setting, minimizing inspiratory effort sensation during flight may prove useful in reducing pilot RTs during complex behavioral tasks. As such, it is reasonable that minimizing inspiratory effort sensation (independent of device resistance) and maintaining end-tidal gas concentrations may improve attentional performance and vigilance in high-performance aircraft pilots during operational settings.

## Data availability statement

The datasets presented in this article are not readily available because the data used in this manuscript are owned the DoD. Data may be available by contacting the corresponding author and pending USAF STINFO approval. Requests to access the datasets should be directed to EK, eli.kelley@us.af.mil.

## Ethics statement

The studies involving human participants were reviewed and approved by the Mayo Clinic Internal Review Board. The patients/participants provided their written informed consent to participate in this study.

## Author contributions

EK, TC, and BJ contributed to conception and design of the study. EK and TC organized the database, performed statistical analyses, and wrote sections of the manuscript. EK wrote the first draft of the manuscript. All authors contributed to manuscript revision, read, and approved the submitted version.
