# Supplementary figures and images for: Inspiratory threshold loading negatively impacts attentional performance
Source: Front Psychol. 2022 Sep 16;13:959515. doi: 10.3389/fpsyg.2022.959515 (PMC9524251; doi:10.3389/fpsyg.2022.959515)

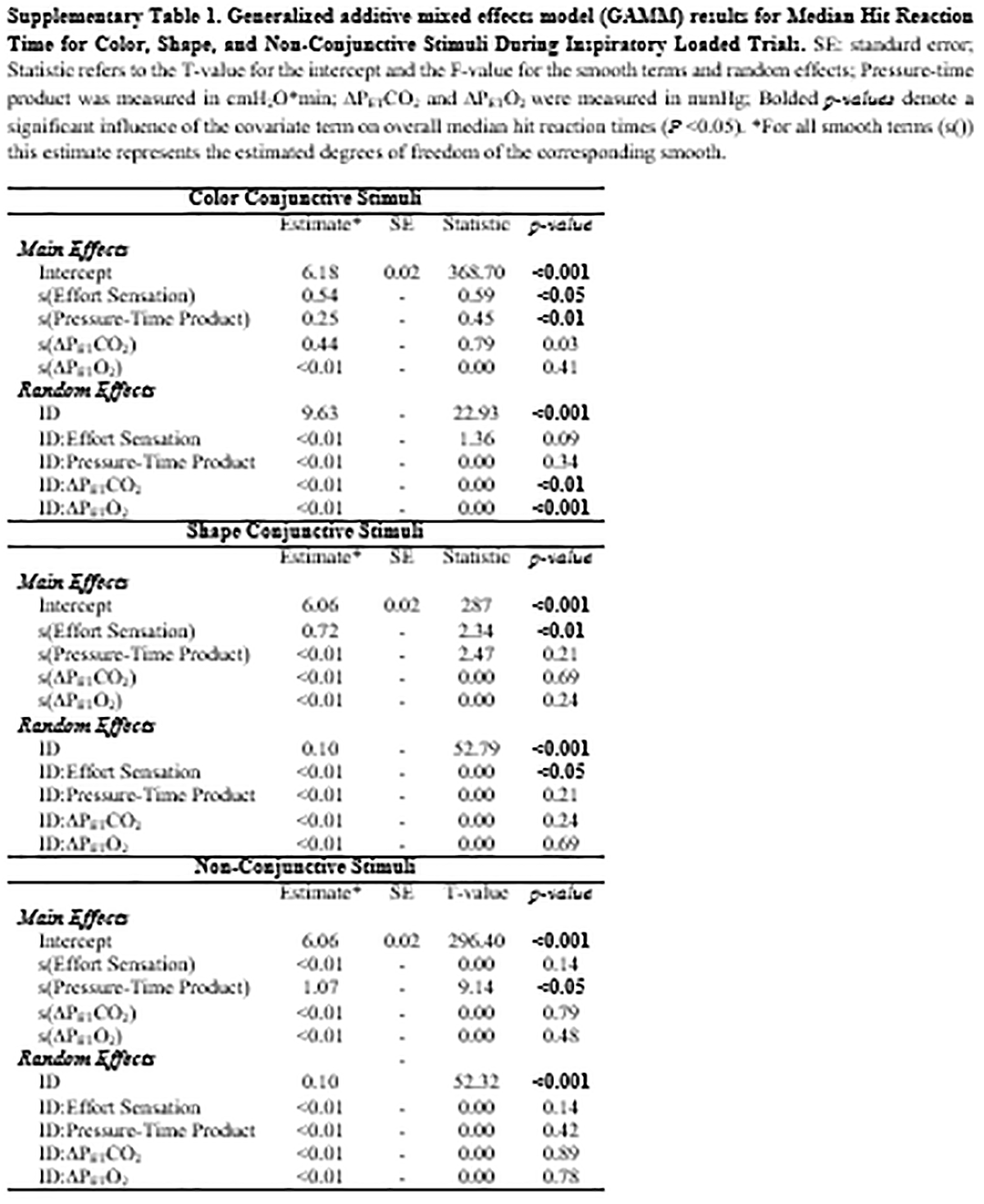

Supplement: Supplementary file 1 [file Image_1.TIF]

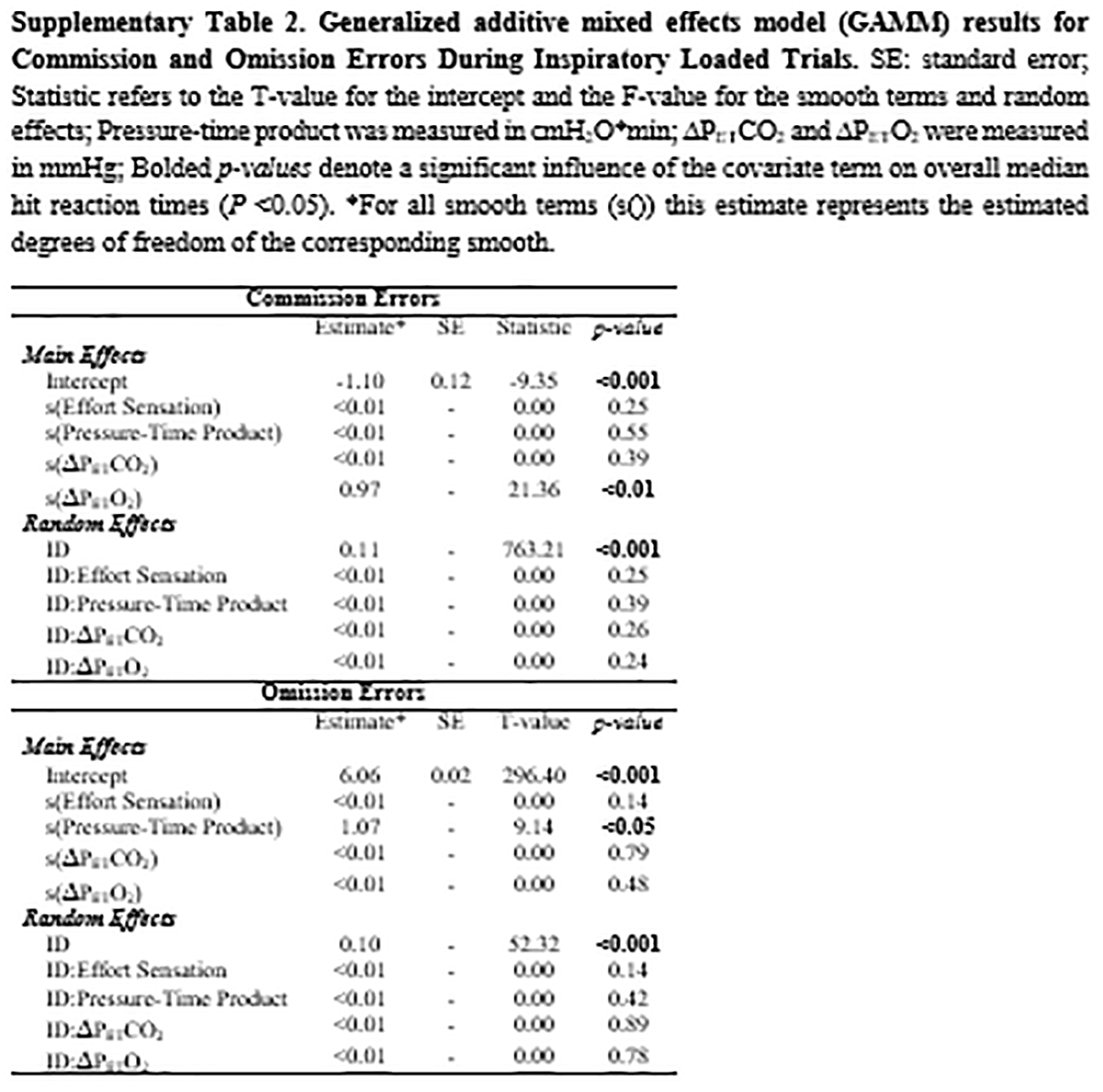

Supplement: Supplementary file 2 [file Image_2.TIF]
